# Evidence of emerging hookah use among university students: a cross-sectional comparison between hookah and cigarette use

**DOI:** 10.1186/1471-2458-13-302

**Published:** 2013-04-05

**Authors:** Tracey E Barnett, Thalia Smith, Ying He, Eric K Soule, Barbara A Curbow, Scott L Tomar, Christopher McCarty

**Affiliations:** 1 Department of Behavioral Science and Community Health, University of Florida, 1225 Center Drive, Gainesville, FL, 32611, USA; 2 Department of Biostatistics, University of Florida, 22 Buckman Dr, Dauer Hall, Gainesville, FL, 32611, USA; 3 Department of Community Dentistry & Behavioral Science, University of Florida, 1329 SW 16th Street, Gainesville, FL, 32608, USA; 4 Bureau of Economic and Business Research, University of Florida, 221 Matherly Hall, Gainesville, FL, 32611, USA

## Abstract

**Background:**

The emergence of hookah is being noted on college campuses and in large U.S. cities and evidence points to an increasing trend for college students. The purpose of this study was to assess hookah use and identify associations with cigarette smoking and demographic factors.

**Methods:**

An intercept sampling method was used at various locations on a large university campus in the southeastern United States, yielding a high participation rate (52%). A total of 1,203 participants completed a computer-aided survey that assessed the use of tobacco products. The sample characteristics were then weighted to match the University population of students enrolled during the same semester. Bivariate (chi-square and t-test) and multivariate (logistic regression) tests of association were conducted to assess differences between cigarette and hookah users.

**Results:**

Hookah smoking exceeded cigarette smoking for both ever use (46.4% vs 42.1%) and past year use (28.4% vs 19.6%). Females and males used hookah at similar rates. Hispanic respondents had the highest prevalence of current use of hookah (18.9%) and cigarettes (16.4%).

**Conclusions:**

As hookah surpasses cigarette use, efforts need to be made to slow the increase in new tobacco products that are attractive to young adults and that pose many of the same health risks as those related to traditional tobacco products. Prevalence of all emerging tobacco products, including hookah, and the relationship with cigarette use needs to be monitored on an ongoing basis.

## Background

Although cigarette smoking among young people is on the decline, the prevalence of hookah (also called waterpipe or narghile) is increasing, particularly on college campuses across the United States. When examining rates, researchers often categorize responses as “ever use” (or lifetime use) or “current use” (defined as use in the past 30 days). Prevalence estimates for lifetime use of hookah by adolescents and young adults range from 10% to nearly 60% (among adolescent cigarette smokers) [1-7] depending on the population characteristics. Hookah use in the past 30 days has been found to be associated with younger age [3,4,8], being male [1,2,5] and white race [3,4]. In one large study aimed at comparing cigarette and hookah use among college intramural, club, and varsity athletes, 2,576 students (29.5%) reported ever trying hookah tobacco smoking and 631 (7.2%) reported hookah tobacco smoking in the past 30 days [8]. College students who had ever used hookah were significantly more likely to be aged 20–21 (34.2%), male (34.2%), and not black (87.8%) [8]. To broaden the research to include multiple institutions, Sutfin et al. [7] sampled college students from eight universities in one U.S. state; 40.3% of the sample reported ever using hookah and 17.4% of the students reported current (past 30 day) hookah use. Many hookah smokers also reported cigarette use [9].

While hookah use appears to be increasing in popularity, research suggests cigarette use is decreasing. According to the 2009 National Survey on Drug Use and Health (NSDUH) [10], full time college students were less likely to be current cigarette smokers (27.1%) when compared to same-aged peers not enrolled in college (40.6%). The prevalence of daily cigarette use among all current smokers aged 18–25 years dropped from 48.1% in 2008 to 45.6% in 2009 [10]. Additionally, while the CDC [11] reports more current cigarette smoking among males (22.8%) vs. females (17.4%) between 18 and 24 years of age, these rates represent a decline from 18 to 24 year-olds in 2005 (males 28.0% and females 20.7%) [11].

During a time in which cigarette use is showing decreasing trends [12], there is reason to be concerned about emerging tobacco products, including hookah, becoming popular among adolescents and college students. A recent press release from the CDC reported that the drop in cigarette consumption is being “offset by increases in other forms of tobacco” [13]. These alternative forms of tobacco need increased attention to prevent cigarette users from simply switching to a different form of tobacco use. Therefore, the purpose of this study was to assess hookah use and to identify associations with cigarette smoking and demographic factors at the University of Florida (UF), a large university in the southeastern United States.

## Methods

### Sample

We intercepted potential student participants at three locations on the UF campus. We selected locations that reached a diverse group of students in terms of major/affiliated college, sex, race, and level in school (freshman to graduate student). Three locations were used approximately 10 different days for 4–6 hours at a time. Each location’s days and times were different week to week. At the conclusion of each day of data collection, the sample was analyzed to ensure appropriate representation of the target population, with a plan to oversample if it appeared that groups were under-represented in the sample.

To the best of their ability, the research assistants approached every 10^th^ person that passed near the area. Access to each location was such that large groups of people would typically not pass by at the same time. For instance, at one location there is a sidewalk that will comfortably accommodate no more than two or three people side by side. We chose this sampling method as a way to force the research assistants not to select respondents based on any visual characteristics, such as their demeanor. Once they reached 75–100 completed surveys, the team moved to another location. The final sample included 1,203 participants.

### Procedure

At each location, we set up five stations with laptop computers. Each computer had the survey instrument loaded into the computer-assisted personal interview (CAPI) program. Research assistants approached prospective respondents and offered them a $5 gift card for their participation. Students who agreed were given a card with a unique identifier; they then went to a station with computers, read and signed a consent form, typed in their identifier code, and handed the card to the research assistant overseeing the stations. Participants were given the gift card after completing the survey. Using this approach, multiple surveys were conducted simultaneously. The study protocol and survey instrument were approved by the UF IRB. Data were collected during the months of November and December, 2009.

### Survey instrument and measures

We developed a computer-based survey instrument to collect information on study participants’ tobacco-related behaviors and demographic characteristics. A thorough literature review of past studies informed the questions included and the instrument was pilot-tested on a group of students prior to administration for the study.

Participants answered demographic questions regarding their sex, race/ethnicity, and age; academic characteristics such as current level in school (undergraduate/graduate), affiliated college (a drop-down menu of all colleges in the university), and full-time or part-time enrollment status. If individuals were not current UF students, they were not included in the study. Although ethnicity and race were asked separately on the survey instrument, when matching back to university registrar data for weighting to ensure representativeness we found that those items were not reported separately in the database. Therefore, for weighting purposes we first ensured that the proportion of Hispanic and non-Hispanic respondents matched the university registrar and then we categorized by students’ race if they were not of Hispanic ethnicity. Furthermore, those identifying as American Indian and Multiracial were combined into the Other race group for statistical testing.

Participants were asked to identify if they had ever used cigarettes or hookah/waterpipe in their lifetime. Participants who reported ever use were then asked separately whether they had used the product in the past 12 months (year) or past 30 days. After identifying which tobacco products participants had used, participants were also asked which product they tried first and the age of first use for each product. Respondents who reported hookah use were also asked to describe the social setting for use, whether they smoked with others or alone, and where they typically smoked hookah.

### Data analysis

To minimize sampling bias, we computed a sampling weight for each student based on ethnicity and race, sex, level of education, and college affiliation. The sampling weight was computed based on the population distribution from the data obtained from the university registrar office for Fall, 2009. The sampling weight for each participant was calculated by dividing the race-ethnicity/sex/academic proportion for that individual in the target population by the corresponding proportion in the sample. Although race and Hispanic ethnicity were measured separately, they were combined for analytic purposes.

Chi-squared tests were used to test the associations between categorical demographic variables and both cigarette and hookah use. Two independent-sample *t*-tests were used to test the difference in mean age between cigarette and hookah users and non-users. Using logistic regression, odds ratios for hookah use by demographics and cigarette use were calculated. The models developed predicted hookah use at all time frames (ever, past year, or current) first with just demographic variables included (Model 1), and then with the same time frame for reported cigarette use (Model 2). These tests were calculated using SAS statistical software v9.2 (Cary, NC).

## Results

We invited 2,328 individuals to participate in the survey; 1,203 agreed and completed the survey, yielding a 51.7% response rate. We also achieved high participation rates among males (45.7%) and non-whites (46.1%). The weighted percentages and unweighted sample characteristics are presented in Table 1.

**Table 1:** Selected demographic characteristics of study participants (N=1203)

**Characteristic**	**N**	**Weighted%**	**Unweighted%**
Age (y)			
18	154	9.3	12.8
19	205	12.5	17.0
20	224	16.4	18.6
21	235	17.7	19.5
22	127	12.5	10.6
23	77	8.5	6.4
24	61	8.1	5.1
25+	120	14.9	10.1
Sex			
Male	550	46.4	45.7
Female	653	53.6	54.3
Hispanic			
Yes	219	13.0	18.2
No	983	86.9	81.8
Race/Ethnicity			
American Indian or Alaska Native	9	1.0	0.7
Asian/Asian American	162	8.2	13.5
Black or African American	185	8.8	15.4
White/Caucasian	649	63.7	53.9
Multiracial	93	7.4	7.7
Other	88	7.2	7.3
Level			
Undergraduate	956	64.9	79.5
Graduate or professional	247	35.1	20.5

Weighted% was compared to registrar data and weighted to represent the enrolled college population during the same semester as data collection.

Unweighted% represents the raw percentages of the sample from which data was collected.

### Prevalence

Hookah and cigarette total prevalence assessed by ever, past year, and current use and comparison between males and females are presented in Table 2. Overall, hookah smoking was more prevalent than cigarette smoking for ever use (46.4% vs. 42.1%, *χ*^2^ (1)=389.7, *p*<.0001) and past year use (28.4% vs. 19.6%; *χ*^2^ (1)=245.3, *p*<.0001 ). While prevalence of current hookah use (9.9%) was similar to that of cigarettes (10.8%), cigarettes were used more often (*χ*^2^(1)=96.8, *p*<.0001 ). Table 2 also presents the sex-specific prevalence rates of cigarette and hookah use. There were no sex differences in hookah smoking for ever, past year, or current use or cigarette smoking for ever or past year. However, current cigarette smoking was twice as prevalent among males (14.7% vs. 7.4%; *χ*^2^(1) = 16.5, *p* < .05) than females.

**Table 2:** Prevalence of use of hookah and cigarettes by sex

		**Total N (%)**	**Male N (%)**	**Female N (%)**	***χ***^**2**^**(1)**	***p***
***Hookah use***						
	Ever use	546 (46.4)*	268 (47.8)	278 (45.2)	0.4	0.529
	Past year use	361 (28.4)*	177 (28.4)	184 (28.4)	0.0001	0.994
	Current use	131 (9.9)	74 (11.9)	57 (8.2)	3.8	0.051
***Cigarette use***						
	Ever use	485 (42.1)	250 (44.9)	235 (33.6)	1.64	0.201
	Past year use	246 (19.6)	140 (22.4)	106 (17.3)	3.37	0.066
	Current use	141 (10.8)*	91 (14.7)	50 (7.4)	12.67	<.001

Note. Percentages represent weighted percentages.

*p<.05 (*χ*^2^ values in results).

Table 3 presents hookah and cigarette use prevalence by race and ethnicity. Participants identifying as Hispanic reported both the highest cigarette and hookah use rates for each time frame of use (ever, past year, and current), followed by whites, Asian, and other. Participants who identified as black/African American reported little cigarette or hookah use across all three time frames of use.

**Table 3:** Prevalence of use of hookah and cigarettes by race/ethnicity

		**White N (%)**	**Hispanic N (%)**	**Black N (%)**	**Asian N (%)**	**Other N (%)**	***χ***^**2**^**(4)**	***p***
***Hookah use***								
	Ever use	305 (53.5)	123 (55.7)	32 (18.4)	57 (35.3)	29 (27.7)	31.86	<0.001
	Past year use	196 (32.6)	95 (41.9)	18 (10.4)	31 (19.3)	21 (10.8)	56.32	<0.001
	Current use	61 (10.4)	44 (18.9)	5 (3.4)	12 (7.5)	9 (3.6)	32.56	<0.001
***Cigarette use***								
	Ever use	265 (47.2)	109 (50.0)	30 (18.8)	58 (35.4)	23 (28.1)	19.13	<0.001
	Past year use	132 (22.9)	62 (27.8)	9 (6.3)	30 (16.9)	13 (4.9)	54.18	<0.001
	Current use	69 (12.0)	38 (16.4)	6 (3.5)	16 (8.7)	12 (4.6)	23.94	<0.001

Note. Percentages represent weighted percentages.

There was no age difference for ever use, but past year users of hookah were younger (M = 21.0) than those who had not used in the past year (M = 22.5) (*t*(1193) = −4.52, *p* < .001). This relationship was also true for current hookah use: current hookah users were slightly younger (M = 22.0) than non-current hookah users (M = 22.2; *t*(1191)= −3.35, *p* < .001). Ever users of cigarettes were slightly older than never users (22.63 vs. 21. 71; *t*(1202) = 2.17, *p* < .05) but no age differences were found for reported past year or current use.

### Age at first use

Among those who reported ever using cigarettes or hookah, the average age of first tobacco use was 16.4 years (SD = 0.16). Cigarettes were more likely to be the first tobacco product tried (58.0%) than was hookah (18.9%). Respondents who reported cigarettes as their first form of tobacco use were more likely to be under the age of 18 at the time of first use (67.9% vs. 43.6%; *χ*^2^ (1) = 13.26, p<.001). Those who reported trying cigarettes first were more likely to be females (65.3%) than males (51.5%; *χ*^2^ (1) = 6.06, *p* < .05). In contrast, those who tried hookah first were more likely to be over 18 at first use (31.2% vs. 10.7%; *χ*^2^ (1) = 21.86, p<.0001). There were no sex differences for respondents who reported trying hookah as their first tobacco product.

### Social setting

Among hookah smokers, the majority (73%) reported only smoking with friends/others and an additional 22% reported usually smoking with friends/others. Only 4% reported usually smoking alone while no one reported only smoking when alone. With regard to location, nearly all (90.2%) reported smoking in a restaurant/café/bar; 85.4% reported also smoking in a friend’s home/apartment; 51.4% reported smoking in their own home/apartment or dormitory; and 7.3% reported smoking in their parents’ home/apartment.

### Hookah use predictors

Regarding dual use, for current cigarette users 38.2% were also current hookah users and slightly fewer current hookah users (35.2%) also reported current cigarette use. Among current hookah users more than one-quarter (28.8%) reported never having smoked a cigarette.

#### Ever use

Compared to the reference group of African American/black participants, those who identified as Hispanic were 5.5 times (CI 3.1,10.0) more likely to report ever using hookah. White participants were also 5.0 times (CI 3.0,8.5) more likely than blacks, and Asian students were 2.4 times (CI 1.2,4.6) more likely to report ever using hookah. In model 2, those who reported ever using cigarettes were 15.7 times (CI 1.6,21.3) more likely to also report ever using hookah. With ever use of cigarettes in the model, Hispanics were 3.9 times (CI 1.9,7.8) more likely than blacks, and white participants were also 3.9 times (CI 2.1,7.2) more likely to ever use hookah. Additionally, when adjusted for ever cigarette use, younger students were more likely to report ever using hookah (AOR 0.9; CI 0.8,0.95).

#### Past year use

As participants increased in age, they were 0.8 times (CI 0.798,0.9) as likely to report using hookah in the past year. Hispanic participants were 6.9 times (CI 3.3,14.1) more likely to report past year use and whites were 4.7 times (CI 2.4,9.2) more likely to use than black participants. Past year cigarette users (model 2) were 11.2 times (CI 7.9,15.9) more likely to report past year hookah use. The addition of past year cigarette use to the model did not change the demographic predictors, younger students were more likely to report past year hookah use (AOR 0.8, CI 0.75,0.87). Also, Hispanics were 4.9 times (CI 2.3,10.7) more likely to use hookah in the past year than black participants. White participants were 3.5 times (CI 1.7,7.1) more likely to use hookah in the past year.

#### Current use

As participants increased in age, they were 0.9 times (CI 0.8,0.94) as likely to report hookah use in the past 30 days. Hispanic participants were 6.9 times (CI 2.2,22.1) more likely and white participants were 3.5 times (CI 1.1,10.7) to report current hookah use compared to black participants. In model 2, current cigarette users were 6.6 times (CI 4.2,10.4) more likely to report current hookah use. Younger participants were also more likely to report current hookah use (AOR 0.8; CI 0.75,0.9). When current cigarette use was included in the model, only Hispanic respondents were 5.5 times (CI 1.7,18.2) more likely to also report hookah use compared to black participants Table 4.

**Table 4:** Predicted odds (AOR) of hookah use by demographics (first) and demographics plus cigarette use

	**Ever use hookah**		**Past year use hookah**		**Current use hookah**	
	**AOR (CI)**		**AOR (CI)**		**AOR (CI)**	
	**Model 1**	**Model 2**	**Model 1**	**Model 2**	**Model 1**	**Model 2**
**Hookah use predictors**						
Age	1.0 (0.95,1.1)	0.9 (0.8,0.95)*	0.8 (0.798,0.9)*	0.8 (0.75,0.87)*	0.9 (0.8,0.94)*	0.8 (0.75,0.9)*
Race/Ethnicity						
Hispanic	5.5 (3.1,10.0)*	3.9 (1.9,7.8)*	6.9 (3.3,14.1)*	4.9 (2.3,10.7)*	6.9 (2.2,22.1)*	5.5 (1.7,18.2)*
White	5.0 (3.0,8.5)*	3.9 (2.1,7.2)*	4.7 (2.4,9.2)*	3.5 (1.7,7.1)*	3.5 (1.1,10.7)*	3.0 (0.9,9.2)
Asian	2.4 (1.2,4.6)*	1.9 (0.9,4.0)	2.2 (0.9,4.9)	1.6 (0.7,3.9)	2.3 (0.6,8.5)	2.0 (0.5,7.9)
Other	1.6 (0.9,3.1)	1.8 (0.8,3.8)	1.5 (0.6,3.5)	1.7 (0.7,4.2)	1.3 (0.3,5.6)	1.4 (0.3,6.0)
African American/Black	ref	ref	ref	ref	ref	ref
Sex						
Male	1.1 (0.9,1.4)	1.0 (0.8,1.3)	1.1 (0.8,1.4)	0.9 (0.7,1.2)	1.7 (1.1,2.4)*	1.4 (0.9,2.0)
Female	ref	ref	ref	ref	ref	ref
*Reported cigarette use^*		15.7 (11.6,21.3)*		11.2 (7.9,15.9)*		6.6 (4.2,10.4)*

*indicate significant predictor.

^Reported in same time frame as hookah.

## Discussion

During a time in which efforts and resources are devoted to reducing tobacco use, some decreases in cigarette smoking may be replaced by alternative tobacco products, such as hookah. At the University of Florida, hookah use has surpassed cigarette use for ever and past year use; while current use rates for both hookah and cigarettes are around 10%.

### Prevalence

Other studies [3,4,7,8] have reported similar rates of hookah use, but hookah smoking lagged behind cigarette smoking in those studies. The overall prevalence for ever use (46.4%), past year (28.4%), and current use (9.9%) are similar to those reported for a University sample in 2008 by Primack et al. [3], who reported ever use at 40.5%, past year use of 30.6% and current use of 9.5%. Grekin and Ayna [2], however, found much lower rates in a university sample with only 15% ever use and 12.4% past year use (this study did not report current use). Eissenberg et al. [4] reported higher rates (ever 48.4%, past year 43.4%, and current 20%) in their college study of Introduction to Psychology students. However, the cigarette rates from the same sample [4] were significantly higher than their hookah rates and they were higher than the national averages for cigarette use with ever cigarette use at 73%, past year use at 57.7% and current cigarette use at 41.5%. Finally, in a sample of eight universities, Sutfin et al. [7] also reported similar ever use prevalence of 40.3%, but a much higher current use of 17.4%. All of these studies indicate popularity of hookah, while the current study shows it may even be more popular than cigarettes.

Though prior studies [1-5,8] found higher rates of hookah use among males, in the present study females and males reported similar rates of hookah use. This finding held even when covariates were added into a logistic regression model to predict hookah use, controlling for age and race differences. Similar trends were found for cigarette use. Again, prior research indicated higher prevalence of cigarette use for males [8,11] across all levels of use, while this study found no sex differences in cigarette smoking rates for ever or past year use, although males surpassed females for current smoking. This is a potential trend to monitor, or there may be something unique with this sample given the high rates of hookah use for females as well.

The racial differences in both cigarette and hookah use in our sample also appeared different from past studies. We found that all levels of hookah and cigarette use (ever, past year, and current use) were most prevalent among Hispanics, followed by whites, Asians, then other. Past research on both adolescent [14] and college samples [15] indicated the highest tobacco prevalence among non-Hispanic whites, followed by Hispanics and lastly African Americans. Similar to the sex differences found in the current study, the higher hookah and cigarette use prevalence among Hispanic participants could be a reflection of this university sample which draws heavily upon students from the Miami area. Although many studies are only able to statistically assess white vs. other race (combining minority respondents), hookah use is typically reported more often for whites [4,5,8,16]. Sutfin et al. [7] found no difference between white and Hispanic hookah prevalence in their sample. These inconsistent trends need to be further assessed in future studies, with enough sampling of minorities to provide detailed information.

### Age

Hookah use was more likely to be reported by younger students. This pattern is also seen in research by Primack et al. [3]. In the present study, current and past year users were found to be younger compared to those who reported not using in the past month or year. The fact that individuals only need to be 18 years of age to enter the relatively large number of hookah venues available in this university town may be influential in this regard.

Age at first use findings indicated differences between cigarette and hookah users as well. Participants who reported trying cigarettes as their first tobacco product were more likely to be under age 18 at first use, while those who reported trying hookah first were more likely to be over age 18 at first use. While tobacco can be purchased and used at age 18 legally, this pattern is of concern as traditionally it was thought that not trying tobacco products by the age of 18 reduced the odds that a young adult would ever become a tobacco user [17]. The pattern of hookah use and its acceptability among these novice users may change the rates of multiple tobacco products used later into young adulthood. Cigarette users were also highly predictive of hookah use, indicating that many young adults already using cigarettes are now using at least two forms of tobacco.

### Social setting

Hookah smoking is a social activity for many adolescent and young adult users. This fact held true in the current study in which the overwhelming majority reported using hookah while with others, which is supportive of previous research regarding the situation in which respondents use hookah [18]. Also supportive of past research [16,18], participants from the current sample indicated cafes/restaurants/bars were the most common place to use hookah, followed by at friends’ houses, further supporting the social nature of this form of tobacco use.

### Predictors of hookah use

There is concern that concurrent tobacco product use might potentially multiply tobacco’s deleterious effects. In this study, 35% of current hookah users also were current cigarette smokers as compared with the 55% found in other recent research [7]. Sterling and Mermelstein [1] reported that adolescent current cigarette users also report a high prevalence of hookah use, and trying cigarettes is associated with using a variety of other tobacco products. In this study, cigarette use was highly predictive of hookah used in the same time frame, even after controlling for demographic characteristics. Surveillance systems need to continue to monitor all tobacco products, including adding hookah and other alternative forms to studies on adolescent cigarette use, to understand the role certain tobacco products play in influencing multiple forms of tobacco use.

While the relationship between cigarette and hookah use is of concern, it is also important to note that of the hookah users, 65% reported *not* being current cigarette users. Over one-fourth (28.8%) of current hookah users reported never having tried a cigarette. This is particularly interesting as it indicates that a substantial number of the participants may have been nicotine naïve had it not been for hookah. Multiple interventions are needed to target dual users, but interventions also need to target the potential myths adopted by hookah users regarding its relative safety, even if they understand the risks in cigarette smoking [5].

### Acceptance and availability

Sutfin et al. [7] demonstrated that the number of hookah venues in a geographical area was related to hookah smoking prevalence, which potentially holds true for this study as well. At the time of data collection, the community surrounding the University of Florida had seven establishments that offered hookah smoking as part business. While there is no comparison community included in this study, college towns appear to be a major target market areas for hookah venues. For example, Primack et al. [19] reported four hookah venues opening within five miles of Carnegie Mellon University and the University of Pittsburgh. Primack et al. [20] also mapped the concentration of 144 U.S. hookah establishments that advertised on the internet. They found that while those establishments were located in all regions of the country, the heaviest concentrations per population were in 11 states, including Florida. Smith et al. [16] reported that adolescents first learned about hookah from friends (50.3%) but also from seeing a hookah lounge (20.9%). The availability and acceptance of this social arena to share tobacco smoking is one possible explanation for the dramatic increase in prevalence observed among adolescents and young adults.

### Limitations

The intercept sampling method remains a form of convenience sampling; however, every 10^th^ student was approached in the effort to reduce systematic bias. Additionally, the data were then weighted to the registrar’s list of currently enrolled students in the same academic semester to further create a more representative sample of the general student population. The prevalence results presented here are also only representative of one university in the southeast region of the United States. In general, the prevalence and patterns of tobacco use vary by locale. Similarly, states differ on policy restrictions for tobacco use in work places, public spaces, restaurants, and bars. However, the present information is helpful for understanding the use of traditional and emerging tobacco products at a major U.S. university.

## Conclusions

Hookah smoking remains a threat to adolescent and young adult populations as its popularity continues to grow, primarily due to widespread availability [7] and lack of restrictions on establishments [21]. In this university town, the availability of hookah is high enough to provide the first known study in which ever use and past year use of hookah surpassed that of cigarette smoking in a U.S. university student population. The prevalence rate for current use of cigarettes and hookah was close and if the availability remains high, hookah may surpass cigarettes in current use as well. This trend not only needs continuous monitoring, but addressing hookah in efforts to reduce cigarette use is warranted. Examples include policy-level restrictions on availability and individual educational efforts for both adolescents and young adults regarding hookah. If openness to hookah leads to more cigarette acceptability, as has been seen in the past with cigar users [22], the tobacco use by adolescents and young adults in the U.S. is poised to increase, perhaps at dramatic rates.

## Competing interests

The authors declare that they have no competing interests.

## Authors’ contributions

TB led the conceptualization and design of the project; TS contributed to the compilation of the current scientific research; YH conducted much of the analysis; EKS contributed to interpretation of data and linking back to the current research; BAC conducted critical reviews at every stage of the project from acquiring grant funding to the final manuscript; SLT also provided critical revision of the final content; CM led the acquisition of data and contributed significantly to the data collection method. All authors read and approved the final manuscript.

## Pre-publication history

The pre-publication history for this paper can be accessed here:

http://www.biomedcentral.com/1471-2458/13/302/prepub
